# Supplementary material for: Unlocking Potential: A Comprehensive Overview of Cell Culture Banks and Their Impact on Biomedical Research
Source: Cells. 2024 Nov 10;13(22):1861. doi: 10.3390/cells13221861 (PMC11593027; doi:10.3390/cells13221861)
Supplement: Supplementary file 1 [file cells-13-01861-s001.zip › cells-3291592-supplementary.pdf]

Supplementary Files

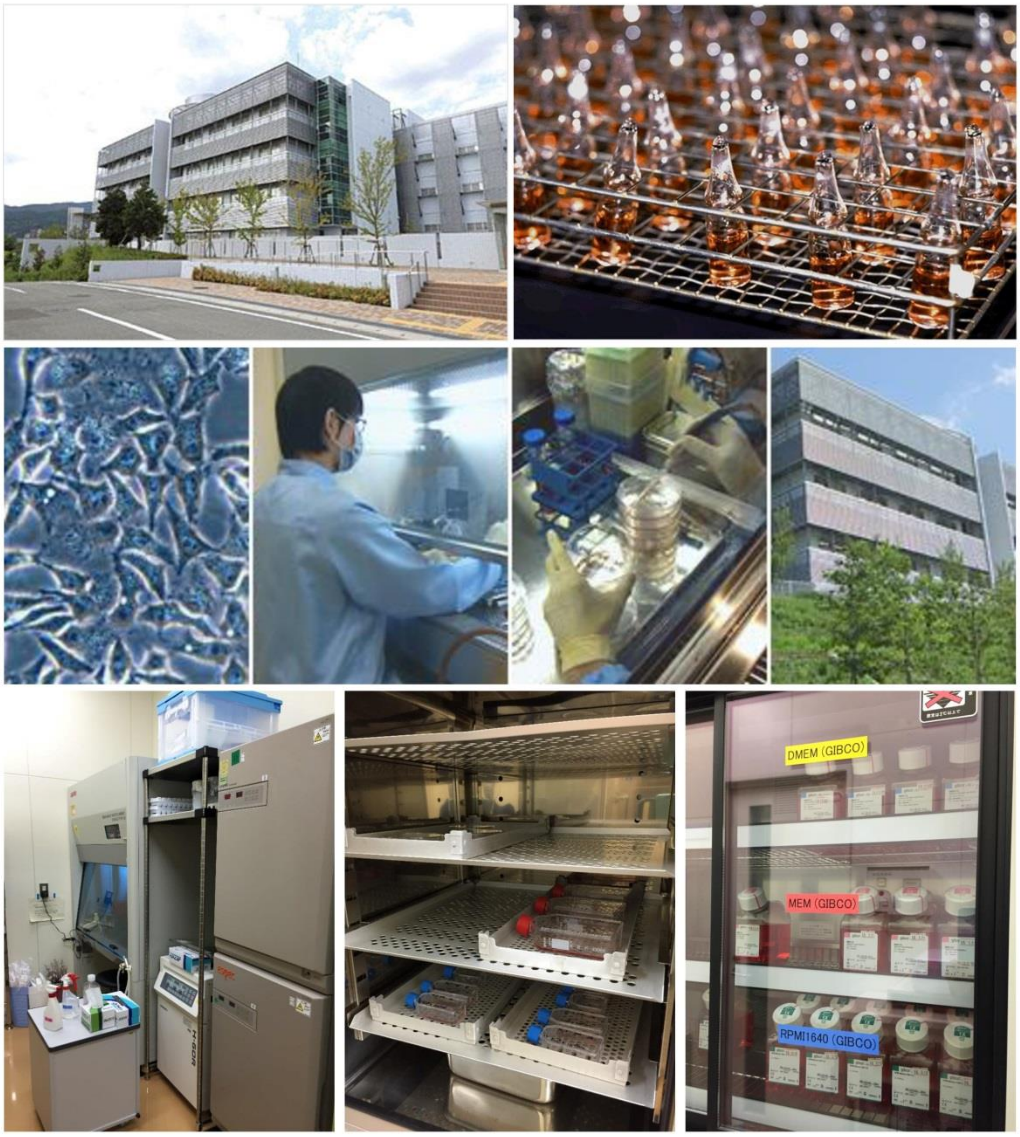

Figure S1. Impressions from the JCRB. The JCRB cell bank is a renowned repository that offers a diverse collection of over 1600 human and animal cell lines, including cancer, genetically modified, and immortalized cells. It focuses on high-quality standards and strict quality control measures to ensure that all distributed cell lines are contaminant-free and authenticated. The bank plays a vital role in supporting cutting-edge research across various fields. The main building of the JCRB is showcased, along with several impressions highlighting the work conducted in its cell laboratories. These images provide a glimpse into the advanced techniques and meticulous processes employed by the dedicated team at JCRB. From cell culture to quality control, every aspect reflects a commitment to excellence and innovation in cellular research.

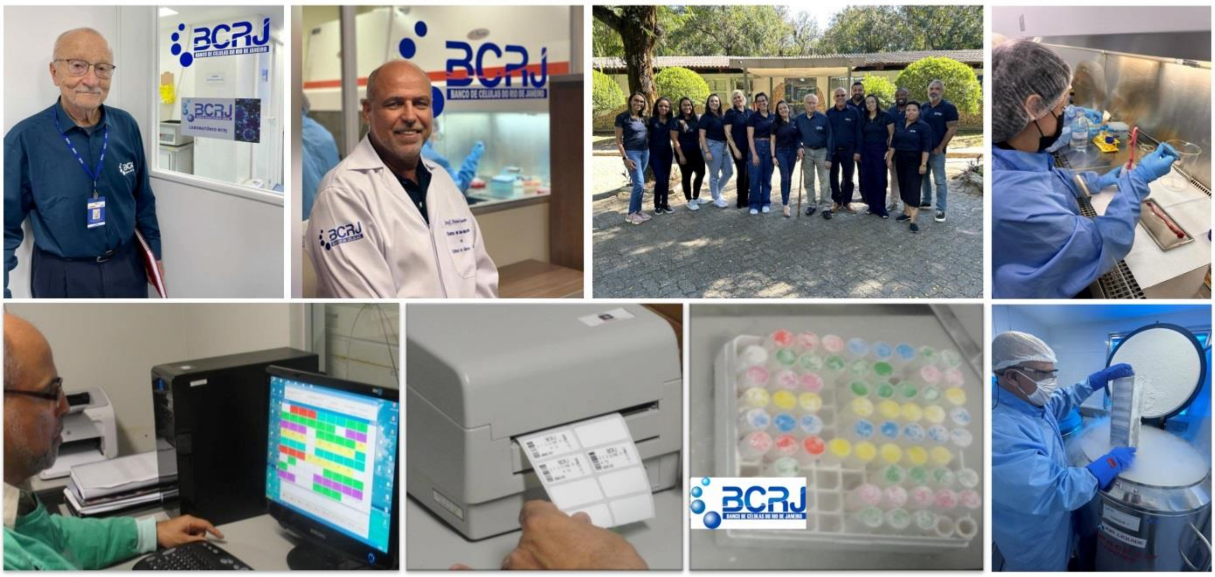

Figure S2. Impressions from the BCRJ. The Rio de Janeiro Cell Bank (BCRJ), founded in 1980 by Professor Radovan Borojevic in collaboration with scientists, technicians, and students from the Federal University of Rio de Janeiro and the Paul Ehrlich Scientific and Technical Association, is a leading center for cell culture in Brazil and South America. It is the largest collection of human and animal cell lines in the continent and the only one providing services in Brazil. Operated by a private, non-profit organization, the BCRJ upholds rigorous standards for its cell lines. Human cell lines are authenticated using the STR technique, while animal-derived cells are verified through DNA barcode analysis. To detect mycoplasma contamination, the BCRJ utilizes PCR and bioluminescence methods. The cells are stored in the vapor phase of liquid nitrogen to preserve their viability. Furthermore, the BCRJ has developed specialized software to efficiently manage its collection and optimize all related processes. The current CEO of the BCRJ is Professor Antonio M. Monteiro.
